# Inhibition of heat shock protein family A member 8 attenuates spinal cord ischemia–reperfusion injury via astrocyte NF-κB/NLRP3 inflammasome pathway: HSPA8 inhibition protects spinal ischemia-reperfusion injury

**DOI:** 10.1186/s12974-021-02220-0

**Published:** 2021-08-06

**Authors:** Jingyi Mi, Yang Yang, Hao Yao, Zhirong Huan, Ce Xu, Zhiheng Ren, Wenfu Li, Ying Tang, Rao Fu, Xin Ge

**Affiliations:** 1 Department of Sports Medicine, Wuxi 9Th Affiliated Hospital of Soochow University, Wuxi, 214000 Jiangsu China; 2 Department of Neurosurgery, Central Hospital of Jinzhou, Jinzhou, 121001 Liaoning China; 3 Department of ICU, Wuxi 9Th Affiliated Hospital of Soochow University, Wuxi, 214000 Jiangsu China; 4 Department of Anatomy, School of Medicine, Sun Yat-Sen University, Guangzhou, 510080 Guangdong China; 5 Department of Microbiology, Biochemistry, & Molecular Genetics, New Jersey Medical School, Rutgers, The State University of New Jersey, Newark, 07103 NJ USA; 6 Brain Research Centre and Department of Biology, Southern University of Science and Technology, Shenzhen, 518055 Guangdong China; 7 Orthopedic Institution of Wuxi City, Wuxi, 214000 Jiangsu China

**Keywords:** HSPA8, Spinal cord ischemia–reperfusion injury, Astrocyte, Neuroinflammation, NF-κB, NLRP3

## Abstract

**Background:**

Astrocyte over-activation and extensive neuron loss are the main characteristic pathological features of spinal cord ischemia–reperfusion injury (SCII). Prior studies have placed substantial emphasis on the role of heat shock protein family A member 8 (HSPA8) on postischemic myocardial inflammation and cardiac dysfunction. However, it has never been determined whether HSPA8 participates in astrocyte activation and thus mediated neuroinflammation associated with SCII.

**Methods:**

The left renal artery ligation-induced SCII rat models and oxygen–glucose deprivation and reoxygenation (OGD/R)-induced rat primary cultured astrocytes were established. The lentiviral vector encoding short hairpin RNA targeting HSPA8 was delivered to the spinal cord by intrathecal administration or to culture astrocytes. Then, the spinal neuron survival, gliosis, and nod-like receptor pyrin domain-containing 3 (NLRP3) inflammasome and its related pro-inflammatory cytokines were analyzed.

**Results:**

SCII significantly enhanced the GFAP and HSPA8 expression in the spinal cord, resulting in blood–brain barrier breakdown and the dramatical loss of spinal neuron and motor function. Moreover, injury also increased spinal nuclear factor-kappa B (NF-κB) p65 phosphorylation, NLRP3 inflammasome-mediated caspase-1 activation, and subsequent interleukin (IL)-1β as well as IL-18 secretion. Silencing the HSPA8 expression efficiently ameliorated the spinal cord tissue damage and promoted motor function recovery after SCII, through blockade of the astrocyte activation and levels of phosphorylated NF-κB, NLRP3, caspase-1, IL-1β, and IL-18. Further in vitro studies confirmed that HSPA8 knockdown protected astrocytes from OGD/R-induced injury via the blockade of NF-κB and NLRP3 inflammasome activation.

**Conclusion:**

Our findings indicate that knockdown of HSPA8 inhibits spinal astrocytic damage after SCII, which may provide a promising therapeutic strategy for SCII treatment.

**Supplementary Information:**

The online version contains supplementary material available at 10.1186/s12974-021-02220-0.

## Introduction

Spinal cord ischemia–reperfusion injury (SCII) is the main complication after surgery of the spine, spinal cord, and thoracoabdominal aorta [1]. SCII pathology is subcategorized into primary and secondary injuries, which cause neurologic dysfunction and may eventually lead to paralysis or paraplegia [2]. The global incidence of SCII was 8.0 to 246.0 cases per million people per year [3]. The high incidence and disability rates of SCII exceedingly reduce patients’ quality of life as well as put a huge burden on society [4]. Despite plenty of basic studies and clinical therapeutic interventions about SCII have been implemented, no ideal curative effect has been achieved [5]. Therefore, it is imperative to investigate the pathological process of SCII and develop a new effective therapy.

Neuroinflammatory response exerts a pivotal role in the secondary injury after SCII [6]. It is well known that traumatic injury to the spinal cord results in blood-spinal cord barrier (BSCB) disruption, axonal damage, neuronal loss, and demyelination, followed by a series of secondary injuries that trigger an enormous inflammatory response in the damaged area [7]. Dysregulated inflammation can spread damage to adjacent tissues, induce neuronal apoptosis and even death, and inhibit axonal regeneration and functional recovery after SCII [8]. Targeting the nod-like receptor pyrin domain-containing 3 (NLRP3) inflammasome exacerbates inflammatory response in SCII [9]. Upon the stimuli, the NLRP3 inflammasome activates and subsequently recruits apoptosis-associated speck-like protein (ASC) and caspase-1, which causes the release of pro-inflammatory cytokines IL-1β and IL-18 [10]. Besides, nuclear factor-kappa B (NF-κB) is a key transcription factor in the inducible expression of inflammatory genes during immunological stress [11]. Suppression of NF-κB-mediated inflammatory response ameliorated the hind limb dysfunction of SCII rats [12]. A study shows that NF-κB-induced oligomerization of NLRP3 with ASC and pro-caspase 1 forms the NLPR3 inflammasome [13]. Upon stimuli to stress, the activated NLRP3 inflammasome cleaves pro-caspase 1 to the mature caspase-1 p20 and subsequently releases pro-inflammatory factors IL-1β as well as IL-18 [13].

Although the molecular mechanisms of SCII have not been fully clarified, it is widely considered that ischemia/hypoxia-induced astrocyte injury plays a crucial role in SCII [14]. Astrocytes are the first responders to the SCII. Reactive astrocytes contribute to glial scar formation after SCII and subsequently inhibit axon regeneration, ultimately blocking neurologic functional recovery [15, 16]. A previous study suggested that the reduction of reactive astrocytes contributed to functional recovery after SCII [15]. Furthermore, inactivation of the NLRP3 inflammasome by BAY 11–7082 or A438079 remarkably attenuated glial fibrillary acidic protein (GFAP) immunoreactivity and finally alleviated the spinal cord damage [9].

Heat shock protein family A [HSP70] member 8 (HSPA8), also called HSC70, is a class of molecular chaperones that plays a key role in the axonal transport of synapsin [17]. A previous study suggested that HSPA8 activated NF-κB signaling through destabilizing the inhibitor of kappaB beta (IκBβ) protein and thereby aggravated the inflammation of synovial fibroblasts in rheumatoid arthritis [18]. Besides, HSPA8 exacerbated the postischemic myocardial inflammation and cardiac dysfunction by NF-κB activation [19]. Blockage of NF-κB pathway efficiently ameliorated myocardial function [19]. Zhu et al. found that HSPA8 was abnormally expressed in rats after SCII, which upregulated after 12 h of reperfusion and downregulated after 24 h [20]. However, the effect of HSPA8 on SCII remains unknown. Therefore, the current study aimed to investigate the effect of HSPA8 on spinal astrocytes following SCII, as well as explore whether HSPA8 was involved in the NF-κB signaling and NLRP3 inflammasome in response to SCII.

## Methods

### Experimental groupings and in vivo gene delivery

Eight-week-old male Sprague–Dawley (SD) rats were purchased from Liaoning Changsheng Biotechnology Co., Ltd. All rats were housed in an environment with a temperature of 22 ± 1 °C, light/dark cycle of 12/12 h, the humidity of 45–55%, and ad libitum access to water and food. All the procedures in animal experiments were performed with the approval of the Animal Care and Utilization Committee of Sun-Yat-sen University, and the number of animal use permits is SYXK 2017–0081. After 1 week for adaption, all rats were randomly divided into four groups: (i) sham operation group (sham group), which received abdominal aorta separation with no clip closed; (ii) SCII-0 h group, which received spinal cord ischemia for 1 h and reperfusion for 0 h; (iii) SCII-12 h group, which received spinal cord ischemia for 1 h and reperfusion for 12 h; and (iv) SCII-24 h group, which received spinal cord ischemia for 1 h and reperfusion for 24 h.

To further explore the effects of HSPA8 on SCII, the experimental grouping was as follows: (1) sham group; (2) SCII group, which received spinal cord ischemia for 1 h and reperfusion for 24 h; (3) SCII + LV-sh-NC group, which intrathecally injected with negative control lentiviral vector (LV-sh-NC) (JTS scientific, Wuhan, China) 3 days before surgery, followed by SCII; and (4) SCII + LV-sh-HSPA8 group, which intrathecally injected with a lentiviral vector encoding short hairpin RNA targeting HSPA8 (LV-sh-HSPA8) (JTS scientific) 3 days before surgery, followed by SCII.

### Establishment of SCII rat models

SCII rat models were established as previously reported [21] with some modifications. Rats were anesthetized with 100 mg/kg pentobarbital sodium and the abdominal cavity was exposed to the abdominal aorta, which ranged from left renal artery to bifurcation of the aorta. The rats were administrated intravenously with heparin (130 U/kg) for anticoagulation 5 min prior to clamping the aorta. Next, the abdominal aorta from the left renal artery to aortic bifurcation was clamped using two bulldog clips, and it was confirmed that distal femoral artery pulsation disappeared after clamping of the arterial clip. Following clamping for 1 h, the arterial clamp was removed to allow reperfusion. The wound was sutured after ensuring that no bleeding or injury to the arteries had occurred. The body temperature of rats remained at 36.5 ± 0.5 °C throughout the procedure. Neurological behavioral scores were evaluated 24 h after reperfusion in rats. All rats were sacrificed under deep anesthesia (200 mg/kg pentobarbital sodium) to harvest lumbar spinal cord tissues of L2–L5 segments.

### Neurological behavioral scores

Postoperative neurological behavior was assessed following the Basso, Beattie, and Bresnahan (BBB) scoring criteria [22] that scores range from 0 to 21 points: 0 points, the hindlimbs were completely paralyzed; 1 ~ 7 points, the hindlimb joints could only move within certain degrees; 8 ~ 13 points, the rat could walk within certain degrees except the hindlimb joints could move; 14 ~ 20 points, rats could use their claws for fine movements; and 21 points, motor function of rats was completely normal. BBB score was calculated in a blinded manner.

### Histopathological assessment

Spinal cord tissues fixed with 4% (w/v) paraformaldehyde were embedded with paraffin, sliced into 5-μm slices, deparaffinized, and rehydrated. Next, slices were stained with hematoxylin–eosin (HE) or Nissl’s staining, as well as dehydrated with ethanol and xylene. After mounting with neutral balsam, slices were taken using light microscopy (BX53, Olympus Corporation) at original magnification × 200 (one optical plane per section). Neurons containing a round or ovoid clear nucleus were customarily taken as normal cells [23]. The number of normal neurons in HE-stained sections was calculated by an experimenter who was blind to the grouping.

### Blood-spinal cord barrier (BSCB) examination

BSCB permeability was evaluated by quantification of extravascular Evans blue after SCII [24]. Briefly, 1.5 mg spinal cord tissue sample was incubated in 1 mL formamide for 24 h at 50 °C. Each sample was centrifuged at 14,000* g* for 30 min at 4 °C to collect the supernatant. Evans blue extravasation was quantified by measuring the optical density of the supernatant at 620 nm.

### Immunofluorescence

Spinal cord tissue slices were deparaffinized and rehydrated. After immersing heated sodium citrate for antigen retrieval, the slices were blocked with goat serum for 15 min at room temperature and then incubated with primary antibodies in PBS at a dilution of 1:50 at 4 °C overnight. These primary antibodies included anti-HSPA8 (A14001; Abclonal, Wuhan, China), anti-GFAP (sc-33673; Santa Cruz, Shanghai, China), and anti-NF-κB P65. After washing with PBS three times, the slices were incubated with Cy3-conjugated goat anti-rabbit (1: 200 dilution in PBS; Beyotime, Shanghai, China; red fluorescence) or FITC-conjugated goat anti-mouse (1: 200 dilution in PBS; Beyotime; green fluorescence) IgG secondary antibody for 60 min in dark at room temperature. 4′,6-Diamidino-2-phenylindole (DAPI) (SL038; Solarbio, Beijing, China; blue fluorescence) was used for nuclear staining. Following mounting with antifade mounting medium, the colocalization of HSPA8 and GFAP and the localization of GFAP or NF-κB P65 were observed using fluorescence microscopy (BX53; Olympus Corporation) at original magnification × 400 (1 optical plane/section). The immunofluorescence intensity was analyzed using Image‑pro plus 6.0 software (Media Cybernetics Inc.). GFAP immunoreactive cells were counted by an experimenter who was blind to the grouping.

### Cell isolation and culture

Primary rat spinal cord astrocytes were isolated from SD rats and cultured according to the Xia et al. method [25]. Briefly, the spinal cord tissues were digested with 0.25% trypsin for 6 min, and the supernatant was further digested in DMEM/F12 (PM150310; Procell, Wuhan, China) medium containing 10% fetal bovine serum (FBS) at 37 °C with 5% CO_2_. The cell suspension was centrifuged at 1500 rpm for 5 min. Finally, the cell pellet was resuspended in a complete medium and then seeded on polylysine-coated cultured plates. Primary astrocytes were identified by their typical morphology and positive immunofluorescence-staining for the specific marker GFAP (original magnification × 200).

### In vitro lentivirus infection and Oxygen-glucose-serum deprivation/restoration (OGD/R) procedure

To archive the adequate HSPA8-overexpression spinal astrocytes, the cells were infected with LV-sh-HSPA8 or its negative control (LV-sh-NC) for 72 h in the 6-well plate (4 × 10^5^ cells per well) in a humidified incubator (HF-100, Healforce, Shanghai, China) with 5% CO_2_ at 37 °C. Subsequently, these cells were subjected to an OGD/R procedure that was performed based on a previous study [25, 26]. Briefly, in the oxygen and glucose deprivation phase, the medium was washed with glucose-free Hanks balanced salt solution and changed to glucose-free DMEM/F12. The cultures were subsequently placed in an anaerobic experimental hypoxia chamber (Stemcell, Beijing, China) containing a gas mixture of 94% N_2_, 5% CO_2_, and 1% O_2_ for 6 h. Thereafter, the cultured cells were transferred to a normal DMEM/F12 medium and incubated in a humidified incubator (HF-100, Healforce) with 5% CO_2_ at 37 °C for 24 h to reach reoxygenation.

### ELISA assay

The levels of interleukin (IL)-1β and IL-18 in the tissue homogenates and astrocyte supernatants were determined using corresponding ELISA kits following the manufacturers’ instructions (USCN Life Science, Wuhan, China).

### Western blot assay

Lysis and protein extraction of spinal cord tissues or astrocytes was performed using the RIPA lysate buffer (P0013; Beyotime). The concentration of the extracted protein was determined by the BCA Protein Assay Kit (P0011; Beyotime). The protein (20–40 µg per lane) was separated on 8–15% SDS-PAGE gel and transferred to polyvinylidene fluoride membrane (IPVH00010; Millipore, Billerica, MA, USA). After blocking with 5% (m/v) skim milk for 1 h at room temperature, the membrane was incubated at 4 °C overnight with primary antibodies in skim milk at a dilution of 1:1000. These primary antibodies were as follows: anti-HSPA8 (A14001; Abclonal), anti-NLRP3 (A5652; Abclonal), anti-ASC (A1170; Abclonal), anti-caspase-1 (A0964; Abclonal), anti-NF-κB P65 (AF5006; Affinity, Cincinnati, OH, USA), anti-phosphorylated NF-κB P65 (p-NF-κB P65) (AF2006; Affinity), anti-IL-1β (A16288; Abclonal), anti-IL-18 (A16737; Abclonal), and anti-β-actin (sc-47778; Santa Cruz). After washing with Tris-buffered saline-Tween 20 (TBST) buffer, the membranes were incubated with horseradish peroxidase (HRP)-conjugated goat anti-rabbit (1:3000 dilution; A0208; Beyotime) or mouse (1:3000 dilution; A0216; Beyotime) secondary antibody at 37 °C for 40 min. The membranes were visualized using a chemiluminescence kit (Shanghai 7sea biotech Co. Ltd.) and analyzed using Gel-Pro-Analyzer 4.0 (Media Cybernetics, Inc.).

### Electrophoretic mobility shift assay (EMSA)

EMSA was used to assess the effect of LV-sh-HSPA8 on NF‐κB activation. Firstly, the nuclear extracts of spinal cord tissues and primary astrocytes were harvested based on the instruction of the Nuclear Protein Extraction Kit (P0027; Beyotime). The protein concentration from nuclear extracts was detected using the BCA Protein Assay Kit (P0011, Beyotime). Finally, the DNA-binding activity of NF-κB P65 was determined using the Chemiluminescent EMSA Kit (BITF282; Viagene Biotech Inc.) as instructed in the manufacturer’s protocol.

### Statistical analysis

All data were shown as mean ± standard deviation and analyzed by GraphPad Prism 8.0 software. The two-tailed unpaired Student’s *t* test was used for comparisons between two groups, and one-way ANOVA was followed by Turkey’s for multiple comparisons. A value of *p* less than 0.05 was considered statistically significant.

## Results

### HSPA8 is highly expressed in rats after SCII

**Fig. 1:**
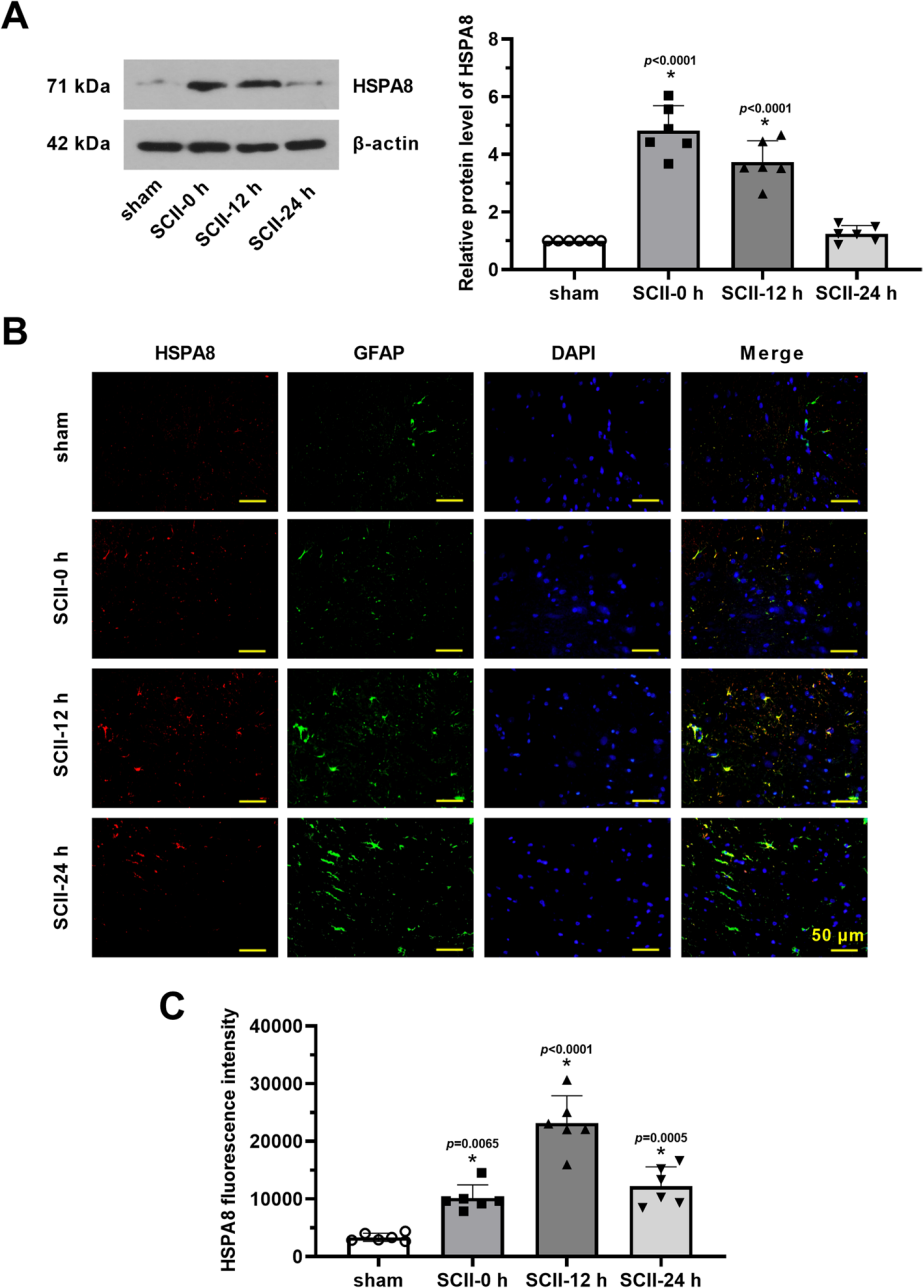
HSPA8 is highly expressed in rats after SCII. **A** The relative protein level of HSPA8 in spinal cord tissues was detected after 0 h, 12 h, and 24 h of reperfusion via western blot assay. **B** Double immunofluorescence of HSPA8 and GFAP in spinal cord tissues was performed. Scale bar = 50 μm. **C** The immunofluorescence intensity of HSPA8 was quantified. These data are representative of three experiments and are shown as the means ± SEM. Data were shown as means ± SD of three independent experiments. *N* = 6 rats per group. Statistical analysis was performed by one-way ANOVA followed by Turkey’s for multiple comparisons. *p < 0.05 vs. sham. SCII, spinal cord ischemia–reperfusion injury; GFAP, glial fibrillary acidic protein

The relative protein level of HSPA8 was upregulated in the spinal cord tissues after reperfusion. However, the increase in HSPA8 was compromised within the following 24 h after SCII (Fig. 1A, p < 0.05). To investigate the effect of HSPA8 on spinal cord astrocytes after SCII, double-labeled immunofluorescence was performed and results showed that HSPA8 and astrocyte-specific marker GFAP were expressed in the spinal cord tissues after reperfusion (Fig. 1B, C, p < 0.05). As depicted in Fig. 1B, the structure merge of HSPA8 and GFAP revealed that HSPA8 expression was closely associated with GFAP after 12 h of reperfusion.

### Knockdown of HSPA8 ameliorates neurological dysfunction and histopathological damage of SCII rats

**Fig. 2:**
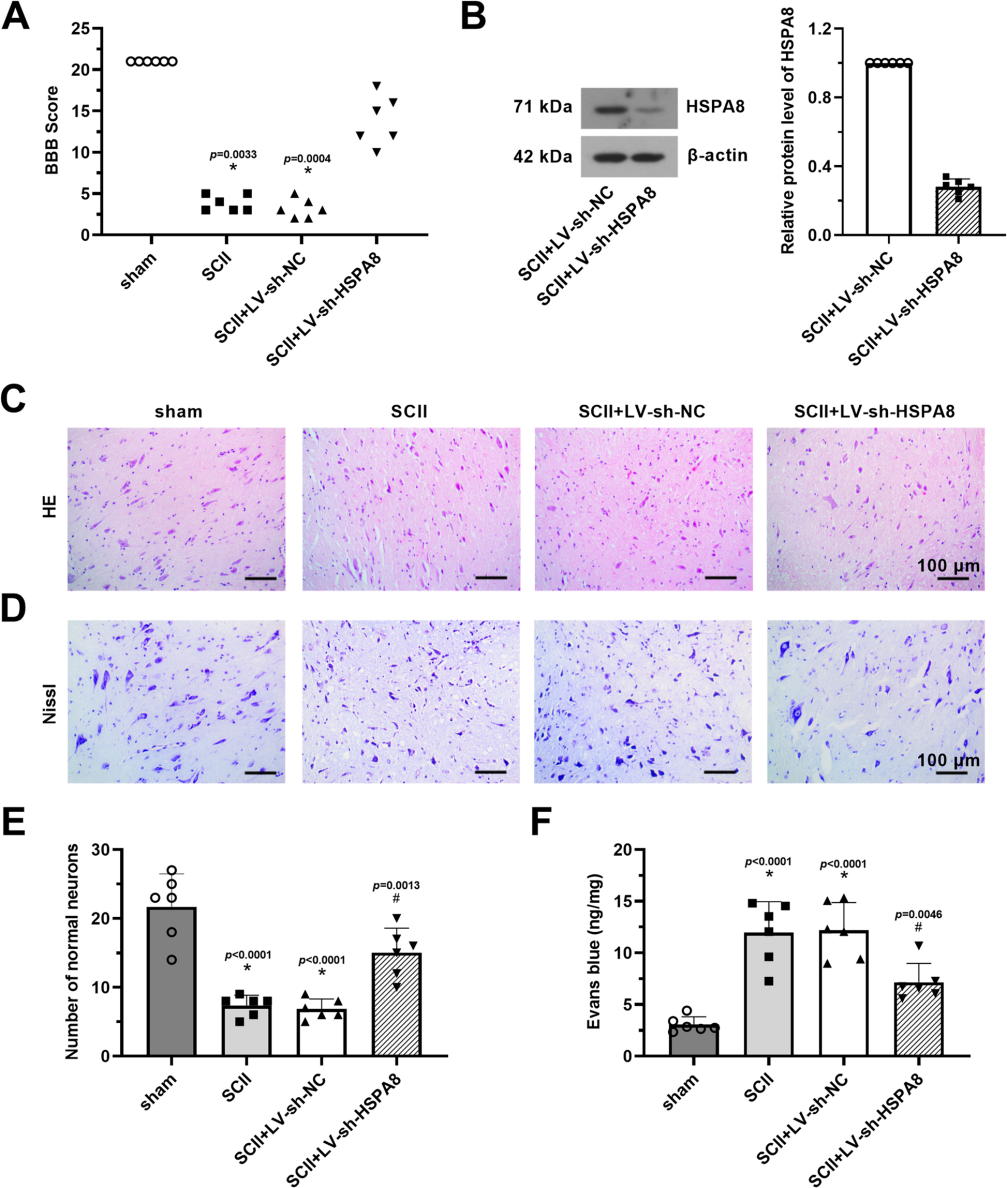
Knockdown of HSPA8 ameliorates neurological dysfunction and histopathological damage of SCII rats. **A** BBB scoring method was used to assess the SCII rats’ neurological function after 24 h of reperfusion. **B** The relative protein level of HSPA8 in spinal cord tissues of SCII rats was detected after injection with LV-sh-HSPA8 via western blot assay. **C** Representative section for HE-stained spinal cord tissues after SCII was assessed. Scale bar = 100 μm. **D** Representative section for nissl-stained spinal cord tissues after SCII was assessed. Scale bar = 100 μm. **E** Quantification of HE-staining normal neuronal number after SCII was analyzed. **F** Quantification of extravascular Evans blue after SCII was analyzed. Data were shown as means ± SD of three independent experiments. *N* = 6 rats per group. Statistical analysis was performed by one-way ANOVA followed by Turkey’s for multiple comparisons (**A**, **E**, and **F**) or two-tailed unpaired Student’s *t*-test between two groups (**B**). *p < 0.05 vs. sham. ^#^ *p* < 0.05 vs. SCII + LV-sh-NC. BBB, Basso, Beattie, and Bresnahan; HE, hematoxylin–eosin; SCII, spinal cord ischemia–reperfusion injury

To determine the neuroprotective effect of HSPA8 inhibition on SCII, rats were intrathecally injected with LV-sh-HSPA8 3 days before SCII surgery, followed by ischemia for 1 h and reperfusion for 24 h. As revealed in Fig. 2A, SCII surgery induced an obvious reduction of BBB score. However, injection with LV-sh-HSPA8 increased the BBB score in rats that underwent SCII surgery (*p* < 0.05). Results of western blot revealed that HSPA8 expression was significantly downregulated in spinal cord tissues of SCII rats after injection with LV-sh-HSPA8, relative to the SCII + LV-sh-NC group (Fig. 2B, p < 0.05). Reduction in normal neuronal number was observed in the gray matter of HE-stained spinal cord tissues after SCII, whereas HSPA8 knockdown had the opposite effect (Fig. 2C–E, p < 0.05). Besides, knockdown of HSPA8 inhibited the reduced number of nissl bodies in spinal cord tissues of SCII rats (Fig. 2D). Quantitative results of extravascular Evans blue revealed that the content of Evans blue was remarkably increased after SCII compared to the sham group, but knockdown of HSPA8 reduced the Evans blue extravasation and thereby protected BSCB integrity (Fig. 2F, p < 0.05).

### Knockdown of HSPA8 inhibits NLRP3 inflammasome activation and phosphorylation of NF-κB in SCII rats

**Fig. 3:**
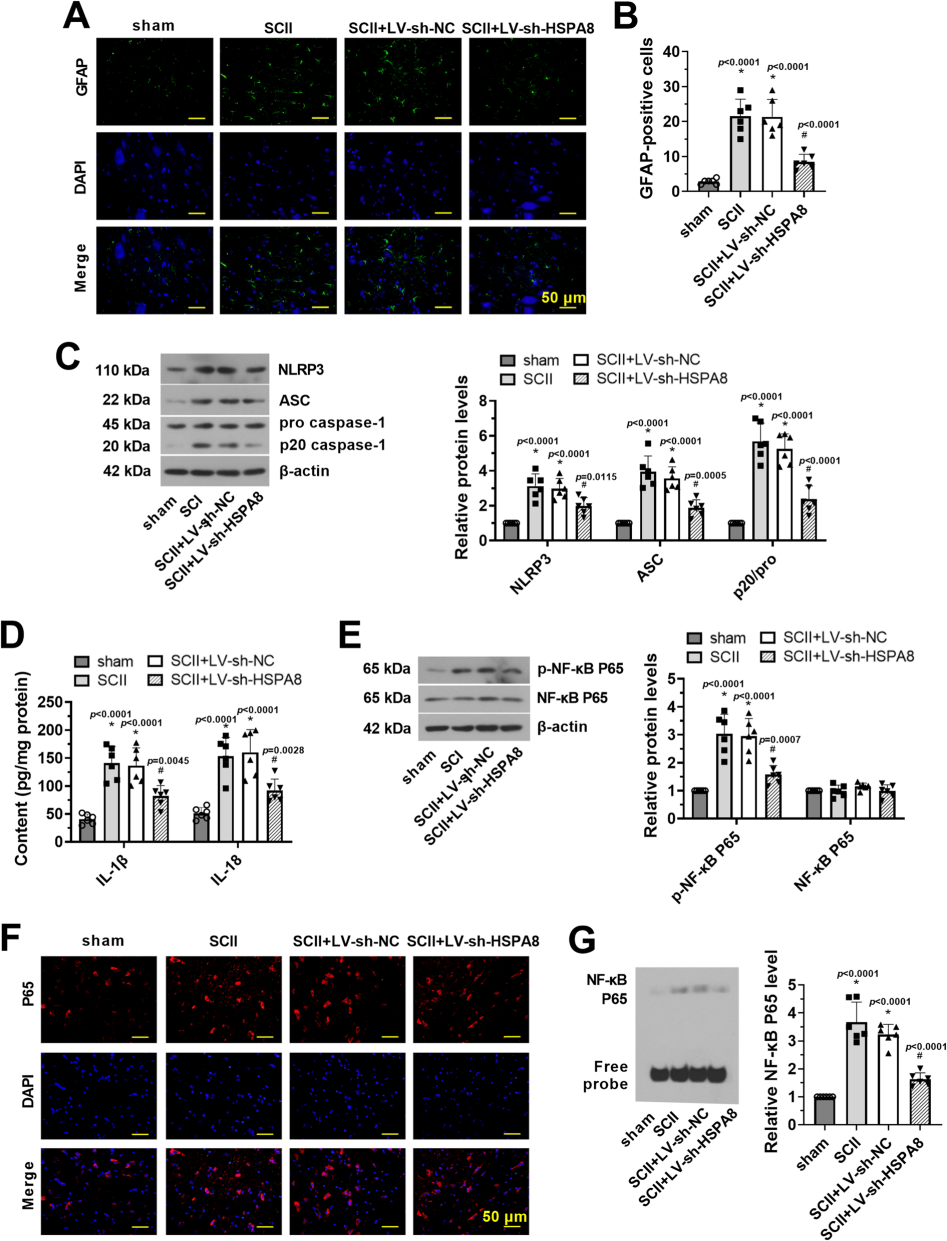
Knockdown of HSPA8 inhibits NLRP3 inflammasome activation and phosphorylation of NF-κB in SCII rats. **A** Immunofluorescence of GFAP in spinal cord tissues after SCII was performed. Scale bar = 50 μm.** B** Quantification of GFAP-positive cells after SCII was analyzed. **C** Relative protein levels of NLRP3, ASC, and p20/pro-caspase-1 in spinal cord tissues of SCII rats were detected after injection of LV-sh-HSPA8 via western blot assay. **D** The contents of IL-1β and IL-18 in the spinal cord tissue homogenate were detected by ELISA assay. **E** Relative protein levels of total NF-κB P65 and p-NF-κB P65 in spinal cord tissues of SCII rats were detected after injection LV-sh-HSPA8 via western blot assay. **F** The intracellular location of the NF-κB P65 in spinal cord tissues of SCII rats was observed by immunofluorescence staining. Scale bar = 50 μm. **G** The DNA-binding activity of NF-κB in spinal cord tissues of SCII rats was revealed by EMSA. Data were shown as means ± SD of three independent experiments. *N* = 6 rats per group. Statistical analysis was performed by one-way ANOVA followed by Turkey’s for multiple comparisons. *p < 0.05 vs. sham. ^#^ *p* < 0.05 vs. SCII + LV-sh-NC. NLRP3, nod-like receptor pyrin domain-containing 3; ASC, the apoptosis-associated speck-like protein containing CARD; SCII, spinal cord ischemia–reperfusion injury; IL-1β/18, interleukin-1β/18; NF-κB, nuclear factor-kappa B

Subsequently, we performed immunofluorescence analysis to examine the protective effect of HSPA8 inhibition on spinal cord astrocytes. As shown in Fig. 3A, B, knockdown of HSPA8 inhibited SCII-induced reactive astrocytes, which was exhibited by the reduction of GFAP-labeled green fluorescence and GFAP-immunoreactive cells (*p* < 0.05). Besides, relative protein levels of NLRP3, ASC, and p20/pro-caspase-1 were significantly decreased in the spinal cord tissues of SCII rats following knockdown of HSPA8 (Fig. 3C, p < 0.05). Decreased levels of IL-1β and IL-18 were also detected in the tissue homogenate of SCII rats after HSPA8 inhibition (Fig. 3D, p < 0.05). Also, we found that the expression of p-NF-κB P65 (ser536) was greatly upregulated in spinal cord tissues after SCII surgery. Knockdown of HSPA8 efficiently decreased the p-NF-κB P65 protein level (Fig. 3E, p < 0.05). Moreover, results of immunofluorescence and EMSA assays demonstrated that knockdown of HSPA8 inhibited the nuclear translocation and DNA-binding activity of NF-κB (Fig. 3F, G, p < 0.05).

### Knockdown of HSPA8 suppresses NF-κB and NLRP3 inflammasome activation in OGD/R-induced primary astrocytes

**Fig. 4:**
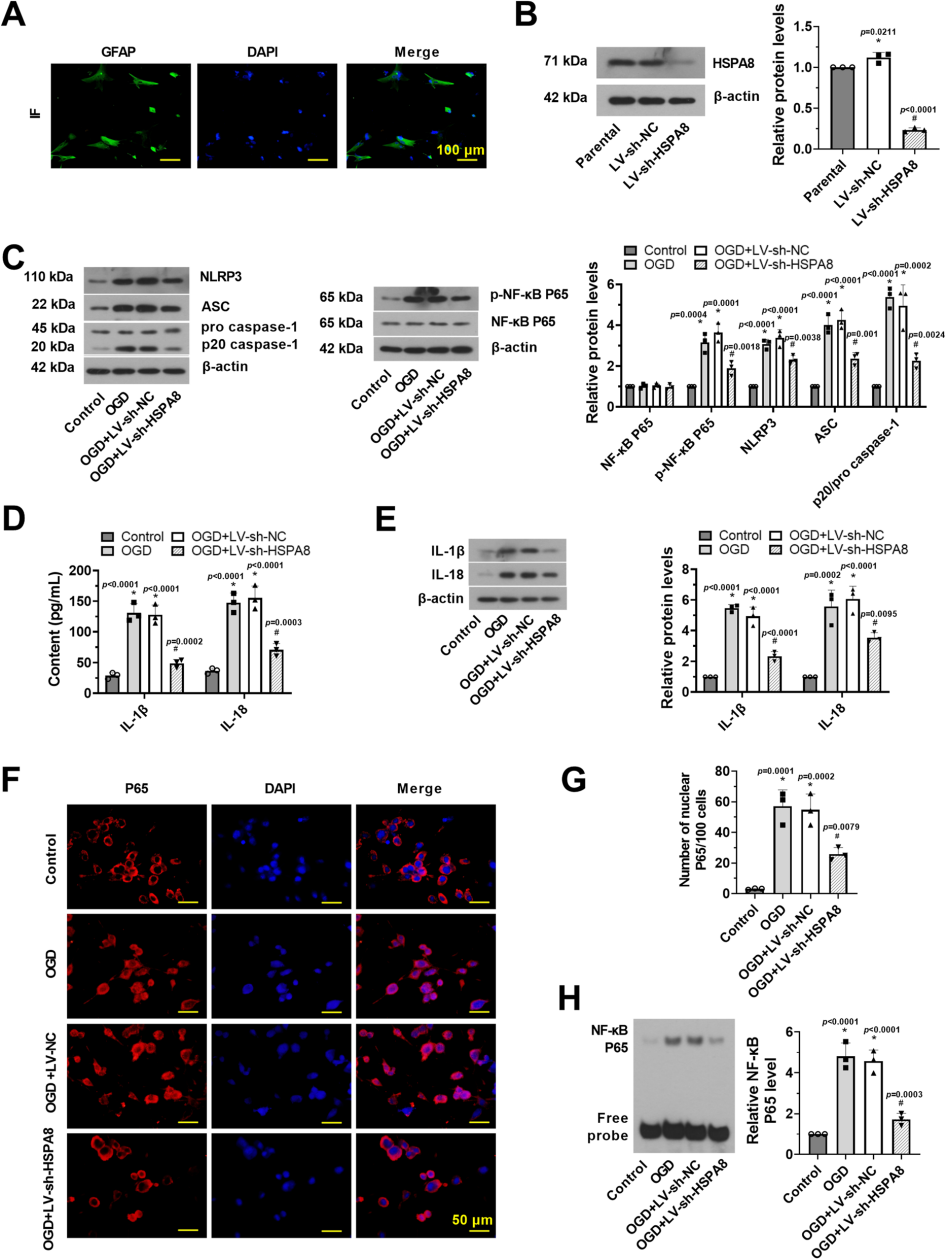
Knockdown of HSPA8 suppresses NF-κB and NLRP3 inflammasome activation in OGD/R-induced primary astrocytes. **A** Immunofluorescence of GFAP in primary rat spinal cord astrocytes was performed. Scale bar = 100 μm. **B** The relative protein level of HSPA8 in primary astrocytes was detected after injection of LV-sh-HSPA8 via western blot assay. **C** The relative protein level of total NF-κB P65, p-NF-κB P65, NLRP3, ASC, and p20/pro-caspase-1 in primary astrocytes was detected after injection of LV-sh-HSPA8 via western blot assay. **D** The contents of IL-1β and IL-18 in primary astrocytes were detected by ELISA assay. **E** Relative protein levels of IL-1β and IL-18 in primary astrocytes were detected via western blot assay. **F** The intracellular location of the NF-κB P65 was observed by immunofluorescence staining. Scale bar = 50 μm. **G** Quantification of NF-ĸB P65-immunofluorescence intensity in the nuclear was analyzed. **H** The DNA-binding activity of NF-κB in primary astrocytes was revealed by EMSA. Data were shown as means ± SD of three independent experiments. *N* = 3 cells per group. Statistical analysis was performed by one-way ANOVA followed by Turkey’s for multiple comparisons. *p < 0.05 vs. sham. ^#^ *p* < 0.05 vs. SCII + LV-sh-NC. NLRP3, nod-like receptor pyrin domain-containing 3; ASC, the apoptosis-associated speck-like protein containing CARD; SCII, spinal cord ischemia–reperfusion injury; IL-1β/18, interleukin-1β/18; NF-κB, nuclear factor-kappa B

Next, to confirm the underlying mechanisms of HSPA8 in SCII, we further examined the effect of HSPA8 in OGD/R-induced primary astrocytes. Results from GFAP-labeled green fluorescence revealed that primary rat spinal cord astrocytes were successfully obtained from SD rats (Fig. 4A). As shown in Fig. 4B, the HSPA8 protein level was substantially decreased in OGD/R-induced primary astrocytes after infection with LV-sh-HSPA8 (*p* < 0.05). Results of western blot and immunofluorescence revealed the phosphorylation and the nuclear translocation process of NF-κB after OGD/R stimulation (Fig. 4C, F, and G, p < 0.05), demonstrating the activation of NF-κB signaling. Levels of NLRP3, ASC, and p20/pro-caspase-1 were also increased in OGD/R-induced primary astrocytes (Fig. 4C, p < 0.05). However, infection with LV-sh-HSPA8 efficiently suppressed NF-κB and NLRP3 inflammasome activation (*p* < 0.05). Next, we detected levels of inflammatory factors (IL-18 and IL-1β) using western blot and ELISA assays. Compared to OGD/R-stimulated astrocytes, astrocytes infected with LV-sh-HSPA8 had lower protein levels of IL-18 and IL-1β (Fig. 4D, E, p < 0.05). Subsequent EMSA analysis suggested that knockdown of HSPA8 efficiently inhibited the DNA-binding activity of NF-κB (Fig. 4H, p < 0.05).

## Discussion

SCII would result in severe motor and functional disorders, as well as even lead to irreversible paralysis. Limiting the development of secondary injury is essential for the recovery of function after SCII, and reactive astrocytes aggravate the secondary injury and inhibit neurologic functional recovery following SCII [27]. The current study found that HSPA8 was highly expressed in the spinal cord tissues of rats after SCII. HSPA8 and astrocyte-specific marker GFAP were closely associated in spinal cord tissues of SCII rats. Genetic knockdown of HSPA8 ameliorated neurological dysfunction, neuron loss, and BSCB permeability in SCII rats. Further in vivo and in vitro evidence determined that the inhibition of spinal reactive astrocytes by HSPA8 knockdown was associated with the inactivation of NF-κB signaling and NLRP3 inflammasome.

HSPA8 is one member of the HSP70 family, which is constitutively expressed in cells [28]. Although HSPA8 shares some of the structural and functional similarities with HSP70, there are some different properties between HSPA8 and the HSP70 family members [28]. Previous studies have shown that overexpression of members of the HSP70 family, such as HSP70 and HSPA12B, exerts a neuroprotective effect under ischemia/hypoxia [25, 29]. HSP70 overexpression inhibits OGD/R-induced apoptosis of bEnd.3 cells [29], and overexpression of HSPA12B protects spinal astrocytes from ischemic injury [25]. In contrast, some research observed that HSPA8 expression was upregulated after cerebral ischemia, and the reduction of HSPA8 contributed to the neurological recovery of the animals [30–32]. Widespread accumulation of HSC70 was found in the brain of patients with multiple system atrophy, which is a progressive neurodegenerative disorder [33]. Consistent with these results, the present study found that HSPA8 was overexpressed in spinal cord tissues of rats following SCII, and the genetic knockdown of HSPA8 exerted a neuroprotective effect on SCII rats. The significant differences between HSPA8 and other HSP70 family members may be associated with their different carboxyl-terminal domains, which participated in mediating substrate specificity and particular biological functions [34].

In neuronal cells, HSPA8 localized predominantly within synapses was associated with synaptic transmission [35]. A previous study has suggested that HSPA8 directly interacts with NF-κB in living hippocampal neurons and inhibition of HSPA8 blockades nuclear translocation of NF-κB [36], which indicated that such direct interactions have an obvious regulatory effect on the resulting signaling and transcriptional regulation. NF-κB P65 is transported from the synapse back to the nucleus by the minus-end motor protein dynein along the microtubule [37]. In this study, knockdown of HSPA8 led to decreased levels of p-NF-κB P65 in spinal cord tissues of SCII rats and primary astrocytes, whereas the level of total NF-κB P65 was no obviously changed after HSPA8 knockdown. Subsequent immunofluorescence and EMSA analyses indicated that knockdown of HSPA8 inhibited spinal astrocyte reactivity after SCII by reducing the transcriptional activity of NF-κB and blocking nuclear translocation. Therefore, we speculate that the nuclear translocation signal might occur as a protein/protein (HSPA8 and NF-κB P65) complex [38]. It has been known that the activated NF-κB translocates to the nucleus and involves the regulation of gene transcription and downstream cellular processes, including cell growth, apoptosis, and inflammation. A previous study suggested that phthalide derivative CD21 alleviated the overactivation of astrocytes by inactivation of NF-κB signaling pathway and NLRP3 inflammasome after cerebral ischemia. Zhu et al. found that wogonoside mitigated SCII-induced neuroinflammation via inhibiting NF-κB and NLRP3 inflammasome activation. In the present study, it was demonstrated that knockdown of HSPA8 ameliorated the inflammation in spinal cord tissues of SCII rats and OGD/R-induced primary astrocytes through the suppression of NF-κB and NLRP3 inflammasome activation.

Considering the HSPA8 expression colocalizes with Iba-1 immunoreactive cells following ischemia (Supplementary Figure), suggesting that the spinal microglial HSPA8 might involve the ischemia–reperfusion injury, which is consistent with the view that spinal microglial cells attribute to the neuron loss associated with ischemia–reperfusion injury. Given that the association between HSPA8 and Iba-1 was weaker than that of HSPA8 and GFAP immunoreactivity cells, especially after 12 h of reperfusion, we emphasize that HSPA8 of the spinal astrocyte might play an essential role in the pathogenesis of ischemia and reperfusion injury. Future study is highly required to further dissect the function of spinal microglial HSPA8 in ischemia and reperfusion injury.

## Conclusion

Collectively, our study shows the neuroprotective effect of HSPA8 knockdown in SCII. HSPA8 inhibition significantly attenuates neuroinflammation and astrocyte overactivation by blocking NF-κB and NLRP3 inflammasome activation. Although additional exploration is still needed, our study demonstrates that HSPA8 is a potential target to prevent SCII-induced astrocytic injury and the HSPA8 depletion might be a promising approach for the treatment of SCII.

## Supplementary Information

**Additional file 1: Supplementary Figure**. HSPA8 is expressed in spinal microglial cells in rats subjected to SCII. Representative immunofluorescence microscope images show that HSPA8 colocalizes with IBA-1(markers of microglial cells) immunoreactive cells in the affected spinal cord tissues. Scale bar=50 μm. SCII, spinal cord ischemia-reperfusion injury; GFAP, glial fibrillary acidic protein. IBA-1, Ionized calcium-binding adaptor molecule-1.

## Data Availability

The data that support the findings of this study are available from the corresponding author upon reasonable request.

## Abbreviations

SCII: Spinal cord ischemia–reperfusion injury
HSPA8: Heat shock protein family A member 8
OGD/R: Oxygen-glucose deprivation and reoxygenation
NLRP3: Nod-like receptor pyrin domain-containing 3
NF-κB: Nuclear factor-kappa B
IL: Interleukin
BSCB: Blood-spinal cord barrier
CNS: Central nervous system
ASC: Apoptosis-associated speck-like protein containing a CARD
GFAP: Glial fibrillary acidic protein
IκBβ: Inhibitor of kappaB beta
SD: Sprague-Dawley
LV: Lentiviral vector
BBB: Basso, Beattie, and Bresnahan
HE: Hematoxylin-eosin
DAPI: 4′,6-Diamidino-2-phenylindole
FBS: Fetal bovine serum
EMSA: Electrophoretic mobility shift assay
