# Supplementary figures and images for: Inhibition of heat shock protein family A member 8 attenuates spinal cord ischemia–reperfusion injury via astrocyte NF-κB/NLRP3 inflammasome pathway: HSPA8 inhibition protects spinal ischemia-reperfusion injury
Source: J Neuroinflammation. 2021 Aug 6;18:170. doi: 10.1186/s12974-021-02220-0 (PMC8349068; doi:10.1186/s12974-021-02220-0)

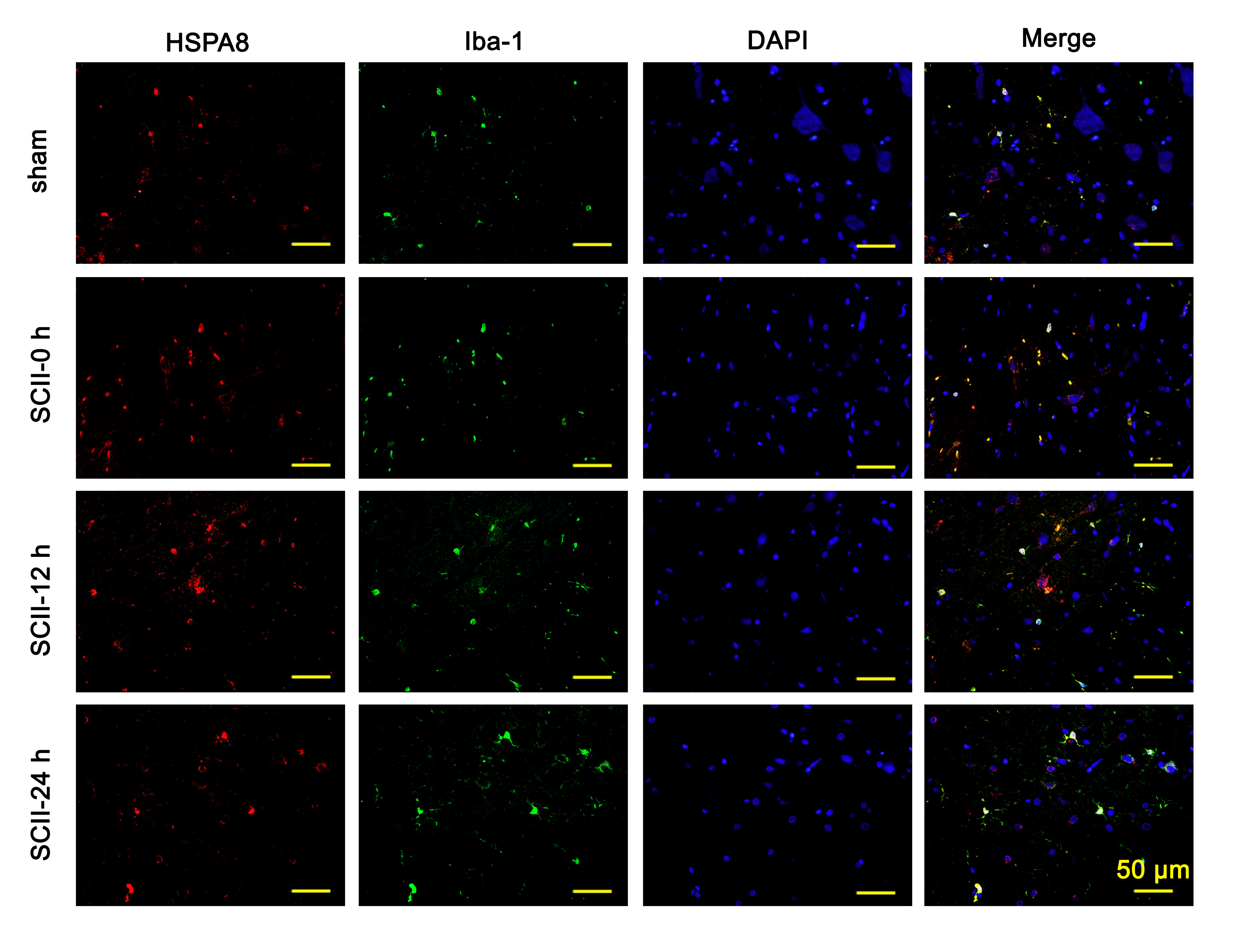

Supplement: Supplementary file 1 — Additional file 1: Supplementary Figure. HSPA8 is expressed in spinal microglial cells in rats subjected to SCII. Representative immunofluorescence microscope images show that HSPA8 colocalizes with IBA-1(markers of microglial cells) immunoreactive cells in the affected spinal cord tissues. Scale bar=50 μm. SCII, spinal cord ischemia-reperfusion injury; GFAP, glial fibrillary acidic protein. IBA-1, Ionized calcium-binding adaptor molecule-1. [file 12974_2021_2220_MOESM1_ESM.tif]
